# School-Family Linked and Online-Offline Combined Tobacco Control Intervention for Adolescents: Protocol for a Cluster Randomized Pilot Trial

**DOI:** 10.2196/86503

**Published:** 2026-10-21

**Authors:** Wanqi Liu, Qi Su, Yuan Cao, Fengyi Huang, Chuning Luo, Xinying Zeng, Ying Liu, Gang Yang, Jinsa Zhou, Meifeng Zhang, Fen Liu, Shiwei Liu

**Affiliations:** 1 Beijing Key Laboratory of Environment and Aging, Department of Epidemiology and Health Statistics, School of Public Health, Capital Medical University, Beijing, China; 2 Chinese Center for Disease Control and Prevention, Beijing, China; 3 Beijing Center for Disease Control and Prevention, Beijing, China; 4 Tongzhou District Center for Disease Control and Prevention, Beijing, China; 5 Fengtai District Center for Disease Control and Prevention, Beijing, China; 6 Fangshan District Center for Disease Control and Prevention, Beijing, China; 7 Chinese Center for Disease Control and Prevention & Chinese Academy of Preventive Medicine, No.27 Nanwei Road, Xicheng District, Beijing, 100050, China, 15101025851

**Keywords:** smoking control, smoking control intervention, Practical, Robust Implementation and Sustainability Model, PRISM framework, protocol, adolescent, behavior change, pilot, implementation

## Abstract

**Background:**

Tobacco use remains a leading preventable cause of disease, yet adolescent-focused tobacco prevention is underexploited. We developed a real-time adaptive intervention that combines school-family collaboration with online-offline delivery (via WeChat mini-programs, games, and curricula) to address adolescents’ overlooked role as health catalysts and persistent household secondhand smoke (SHS) exposure.

**Objective:**

This study aims to evaluate the feasibility and effectiveness of this school-family linked and online-offline combined tobacco control intervention in improving adolescents’ tobacco-related knowledge, attitudes, and behaviors, reducing student smoking prevalence and household SHS exposure, and assessing implementation process indicators (fidelity, participation, and satisfaction) to inform future scale-up.

**Methods:**

This is a cluster randomized pilot trial guided by the PRISM (Practical, Robust Implementation and Sustainability Model) framework. We recruited approximately 1280 seventh-grade students (aged 12‐13 years) from 9 secondary schools across 3 districts of Beijing (urban, peri-urban, and suburban). Classes were the unit of randomization, allocated 1:1:1:1 to a control group (standard education) and 3 intervention arms (online only, offline only, and combined online-offline). The 7-component intervention (lectures, pamphlets, microvideos, peer games, knowledge competitions, online quizzes, and a digital mini-game) was delivered through a school-family digital platform. The coprimary outcomes are changes in tobacco-related knowledge and attitudes, along with implementation process indicators (fidelity, participation, and satisfaction) and feasibility; and the secondary outcomes are tobacco-related behaviors (smoking prevalence and quit attempts) and household SHS exposure. A mixed methods evaluation based on PRISM assessed recipient, provider, and health system levels.

**Results:**

Baseline data collection was completed in November 2024. Data analysis began in late 2025, with final results expected in 2026. The findings will determine whether the intervention improves adolescents’ tobacco knowledge and attitudes, reduces smoking and household SHS exposure, and provides insights into implementation fidelity and satisfaction across delivery modes.

**Conclusions:**

This pilot trial will establish the feasibility and preliminary effectiveness of the intervention. If successful, it may mobilize school-family partnerships using widely available digital platforms to support sustained tobacco control among adolescents and reduce household SHS exposure.

## Introduction

### Background

Tobacco smoking is a leading cause of preventable illness and death worldwide, with more than a billion smokers. Without increased efforts to quit, at least half will die from tobacco-related issues. Smoking also significantly reduces quality of life. Nicotine affects the brain’s reward system, causing addiction, which leads to continued use despite negative consequences. Effective psychosocial and pharmacological treatments have been proven to help people quit, but since most smokers relapse, it is crucial to expand the reach of current interventions and develop new ones [1].

China is one of the largest tobacco consumers in the world. In 2016, the Chinese Government’s “Healthy China 2030” agenda set a concrete target of reducing the overall smoking prevalence among people aged ≥15 years to 20% by 2030 [2]. Achieving this reduction requires widespread cessation among adult smokers and preventing new uptake among adolescents. In China, preventing tobacco use among youth is a key focus of tobacco control efforts. The government has implemented various measures, including incorporating tobacco control into health education curricula, enforcing smoking bans in schools, encouraging smoke-free homes, and running advocacy campaigns [3]. Adolescents are in a critical period of extensive individual cognitive development, and they tend to be curious, show a wide range of interests, and are inclined to emulate those around them [4,5]. The study by Lin et al [6] demonstrated that the number of smoking family members, classmates smoking, the perception that smoking is cool and attractive, and attitudes toward tobacco control policies were the predictors of current smoking behavior (*P*<.05). Adolescent smoking is strongly associated with having parents and friends who smoke [7].

Tobacco control interventions within schools and families have therefore become crucial. However, to date, there is limited evidence regarding the effectiveness of interventions that integrate both school-based and family-based tobacco control measures for adolescents.

Intervention research on adolescent substance use includes curriculum interventions focusing on cognitive behavioral skill enhancement, exercise interventions, peer interventions, family-school cooperation interventions, and electronic interventions. Schools can use equipment and human resources to provide various intervention measures for adolescents, but they also face challenges such as stigmatization, ineffective coordination of multiple resources, and poor implementation effects [8]. A recent review concluded that, due to a lack of research, smoking prevention interventions for children and adolescents involving parental participation seem to be effective, but the overall quality of evidence is moderate. No conclusions can be drawn regarding the impact of parental involvement in interventions on children’s smoking prevention, and the equity aspects of these interventions still require further study [9]. Another study reported the short-term effectiveness of school-based interventions in reducing and preventing adolescent tobacco use.

In terms of intervention channels, smartphones are rather popular in China. As of June 2023, China’s internet populace reached 1.079 billion [10]. Compared with the existing smoking cessation services in China, online interventions can be accessed at any time and from anywhere through a smartphone [11]. Mobile health (mHealth) interventions are increasingly being designed to facilitate health-related behavior change [12].

This suggests that future research should prioritize long-term strategies, integrating digital tools, family, and community involvement, to sustain behavioral changes and effectively combat the tobacco epidemic [13].

Therefore, we designed a cluster randomized controlled trial targeting first-year middle school students from 9 schools in 3 areas of Beijing, China (urban, peri-urban, and rural), aiming to assess the effectiveness of a school-family collaborative tobacco control intervention during its implementation process. The study was named “School-family linked and online-offline combined tobacco control intervention.” We believe that conducting formative evaluation prior to the project intervention, assessing the implementation process during project execution, and evaluating the effectiveness and sustainability after project completion can provide a valuable starting point for planning tobacco control programs to prevent or manage tobacco use among students and enhance their awareness of tobacco.

This pilot trial has 2 integrated objectives. First, guided by the PRISM framework, we aim to evaluate the implementation process of the multicomponent intervention—specifically fidelity, participation, satisfaction, and contextual factors that facilitate or hinder implementation—to inform future refinement and scale-up. Second, we aim to provide preliminary estimates of the intervention’s effects on adolescents’ tobacco-related knowledge, attitudes, and behaviors (coprimary exploratory and secondary outcomes, respectively) across 3 delivery modes (online only, offline only, and combined online-offline), with the expectation that offline and combined modalities will show stronger effects on correcting specific misconceptions (eg, thirdhand smoke harm and e-cigarette addictiveness) due to face-to-face engagement, whereas online delivery may be more effective for broad knowledge dissemination (eg, secondhand smoke [SHS]–related disease cognition), given its wider reach and convenience. However, as this is a pilot trial, effect estimates for behavioral outcomes will be interpreted cautiously and used to generate hypotheses for future research.

### Theoretical and Operational Model

The PRISM (Practical, Robust Implementation and Sustainability Model) framework, an extension of the RE-AIM (reach, effectiveness, adoption, implementation, and maintenance) framework, integrates concepts from social ecology, the PRECEDE-PROCEED model, and quality improvement models [14]. It aims to guide researchers in systematically examining the complex influence of the dynamic context on intervention implementation [15]. This framework possesses the attributes of both a determinants framework and an evaluation framework [16] and is applicable across all stages, from research planning to maintenance. PRISM comprises 4 core dimensions: the intervention (recipients and organization), the recipients (recipients and organization), the implementation and sustainability infrastructure, and the external environment [16].

Since 2008, PRISM has been widely applied across diverse topics, populations, and settings [17]. Research demonstrates that using PRISM to analyze the implementation context can effectively identify barriers to and facilitators of program implementation. This aids in developing or adapting implementation plans and enhances the likelihood of program success [18,19]. While sharing similarities with other implementation science frameworks focused on context (eg, i-PARIHS and NPT) in conducting multilevel assessments (intervention, recipients, and environment), PRISM’s advantage lies in its more focused assessment approach. It prioritizes key factors likely to influence outcomes rather than attempting to be all-encompassing. This focus is particularly crucial for promoting health equity and the institutionalization of research findings [20]. Notably, approximately 78% of studies applying PRISM have incorporated health equity considerations to some extent [17]. This is largely attributable to its mechanism of engaging multiple stakeholders throughout the research process in plan development and adaptation while integrating evaluation consistently [21].

The PRISM framework is the core methodological feature that distinguishes this study from the majority of existing tobacco control intervention protocols.

At the design stage, the 4 PRISM dimensions—intervention, recipients, external environment, and implementation and sustainability infrastructure—were directly operationalized into the activity planning framework. For example, in the offline arm, knowledge lectures and peer games correspond to the intervention dimension; school-family linkage, which expands the target from students to parents, corresponds to the recipients dimension; the selection of 3 developmentally distinct regions and the protocol’s inclusion of community-level environmental assessments correspond to the external environment dimension; and the homeroom teacher supervision mechanism and student assistant training correspond to the implementation and sustainability infrastructure dimension. This framework-driven approach ensures that the intervention content is more systematic and comprehensive than conventional experience-based program design.

At the evaluation stage, the PRISM framework guided the parallel design of both process and outcome evaluations. The semistructured interview guides for the process evaluation were structured precisely according to the 4 PRISM dimensions, allowing specific issues raised by interviewees, such as “rushed activity schedules,” “uncooperative parents,” or “crowded venues,” to be rapidly mapped onto their corresponding framework dimensions. This enables the formulation of targeted improvement strategies (eg, adjusting timing, enhancing communication, or securing better facilities) rather than generic recommendations to “strengthen management.” Similarly, the outcome evaluation questionnaire items were designed to align with the 4 PRISM dimensions, ensuring that the final analytical results can be interpreted in relation to the framework, answering not only whether the intervention worked but also on which dimension it was most or least effective.

As emphasized by Glasgow et al [22] in their historical review of PRISM, the core strength of the framework lies in its capacity not merely to evaluate “outcomes” but to explain “why a given outcome occurred” and “under what conditions.” This protocol is a practical instantiation of this methodological proposition—the anticipated findings are expected to answer not only the statistical question of “whether it works,” but also the implementation science question of “how it works in the real world.”

## Methods

### Study Design

This trial protocol was developed in accordance with the SPIRIT 2025 statement (Checklist 1). This study is a cluster randomized controlled trial aimed at improving tobacco-related knowledge and attitudes, preventing smoking initiation among adolescents, and reducing SHS exposure within households (Figure 1). The study participants were middle school students who received at least 6 months of the school-family linked and online-offline combined tobacco control intervention. After ethics committee approval, recruitment started on July 24, 2024. By June 15, 2025, a total of 1330 middle school students had been recruited. The study lasted for 1 academic year (approximately 7 months) and had reached the required sample size. Due to the randomized controlled trial nature of the study, interventions aimed at enhancing tobacco-related health awareness and preventing smoking uptake were provided. The school was not used as the randomization unit; instead, classes were the unit of randomization. Within each school, classes were allocated to the control group and the intervention group at a ratio of 1:3. Patients and the public were not directly involved in the design, conduct, or reporting of this trial.

**Figure 1.**
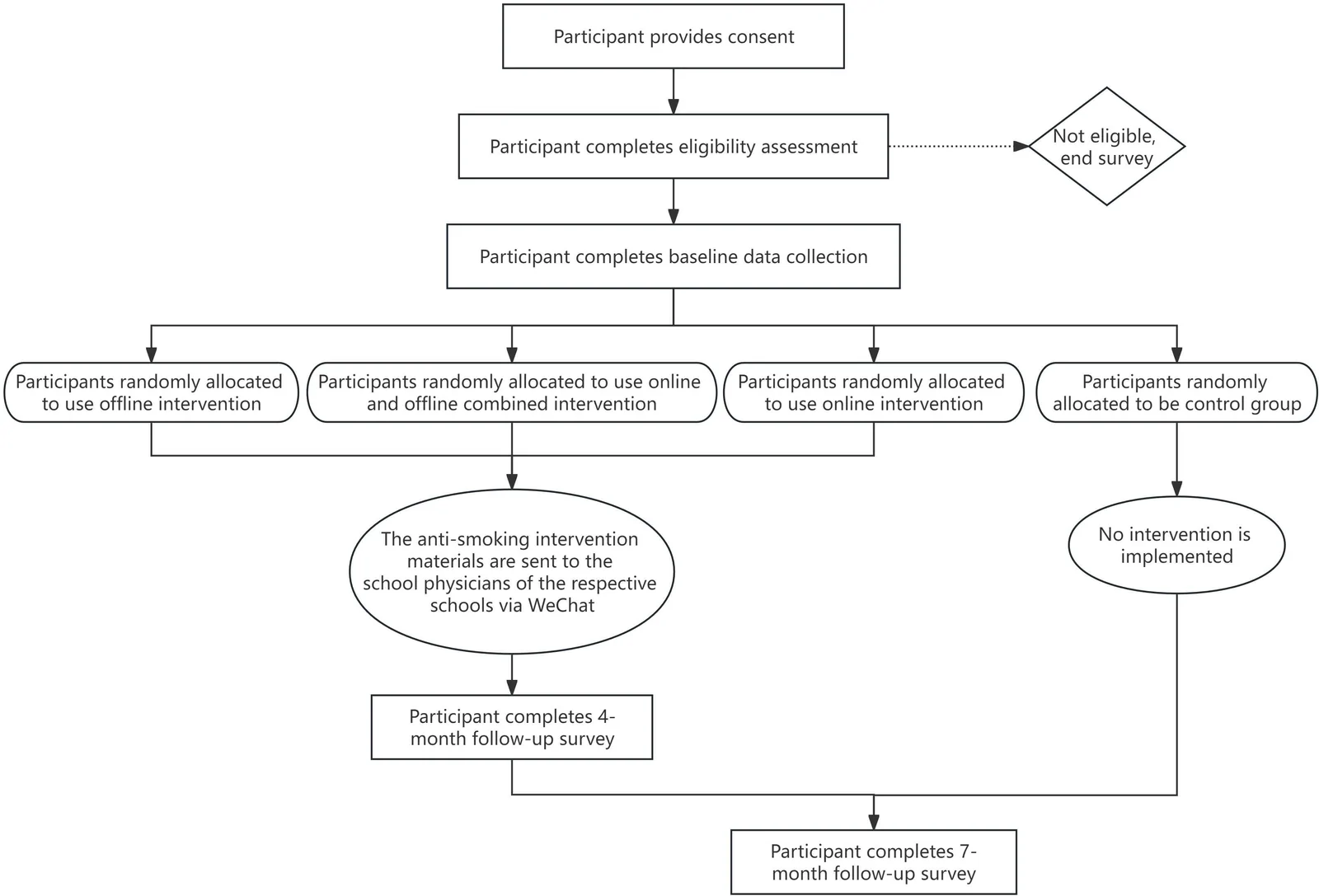
Flow of participants through the study.

### Setting

The study was conducted in Fengtai District, Tongzhou District, and Fangshan District of Beijing, China. These 3 sites represent the urban, peri-urban, and suburban contexts.

#### Study Clusters

We recruited 9 schools from the abovementioned 3 sites, with 3 schools from each area. We included public schools, provided that they (1) had seventh-grade classes, with 30 to 50 seventh-grade students (aged 12‐13 years) per class and (2) school administrators agreed to cooperate.

We excluded schools if they had already participated in other projects and had a busy schedule, making it difficult to cooperate.

#### Study Participants

We recruited 1280 first-year junior high school students (aged 12‐13 years) after obtaining parental consent and student assent through the schools, with at least 30 students from each class. We believe that this grade level has a manageable academic load and that this age group is the optimal one for understanding the information and working with their family members to implement smoking control measures. We included the following students: studying in the first year of junior high school at the participating school, aged between 12 and 13 years; and both the student and the parents agreed to the student’s participation in the project. We excluded the following students if they had any of the following conditions known to the school: physical or mental disabilities leading to long-term absenteeism or leave; learning difficulties and/or special learning needs; and behavioral problems and/or conduct disorders.

### Cluster Randomization and Allocation

Once the necessary baseline data had been logged into the database, the 4 classes from each of the 3 participating schools were randomly allocated to 2 groups—the intervention group and the control group. One class was in the control group, while 3 classes were in the intervention group. The 3 classes in the intervention group were then further divided into 3 subgroups: online intervention group, offline intervention group, and combined online and offline intervention group. The number of classes in each group was equal (ie, 3 classes per group). The intervention group classes from each school were paired with the control group classes for follow-up purposes.

This study used a cluster randomized design with the class as the randomization unit and an allocation ratio of 1:1:1:1 (control:online intervention:offline intervention:combined online-offline intervention). The RAND() function in Microsoft Excel 2021 (version 16.0) was used to generate a sequence of random numbers for class allocation. The specific steps were as follows:

1. Three middle schools were selected from each of 3 districts in Beijing: Fengtai (urban), Tongzhou (peri-urban), and Fangshan (suburban). In each school, 4 classes from the seventh grade were selected.
2. In Excel, the 4 classes in each school were assigned numbers 1, 2, 3, and 4 separately (eg, classes 1‐4 in the Fengtai school; classes 1‐4 in the Tongzhou school; classes 1‐4 in the Fangshan school).
3. The RAND() function was used to generate a random number (range 0‐1) for each class.
4. The classes were sorted in ascending order based on the random numbers.
5. According to the sorted order, the class with the smallest random number was assigned to the control group. The remaining 3 classes were sequentially assigned to the online intervention group, the offline intervention group, and the combined online-offline intervention group.

This process was independently repeated in each school to ensure random allocation of the 4 classes within every school. The random sequence was generated by a statistician on the research team who is not involved in participant recruitment or intervention delivery. The allocation sequence is recorded and sealed immediately after generation and was not revealed until the start of intervention allocation.

This study used a single-blind design. Researchers responsible for delivering lectures and organizing activities, as well as classroom teachers, could not be blinded because the implementation modalities differ inherently across the intervention groups (online, offline, and combined). They were aware of the full group allocation to ensure correct delivery of the interventions.

Student participants were blinded. During the study period, students were not informed that the study involved comparisons between different intervention arms, nor were they told which specific intervention mode (eg, online, offline, or combined) their class belonged to. All intervention activities were conducted under the common name of “Health Education Courses” or “Health Promotion Activities” within the respective classes, to avoid expectation bias or behavioral changes resulting from participants’ knowledge of group assignment.

### Risk of Contamination

A key methodological concern in cluster randomized trials with classes allocated within the same school is the risk of contamination; participants in the control group may be indirectly exposed to intervention components through peer-to-peer communication, shared campus environments, or teacher interactions. Such contamination would bias effect estimates toward the null, potentially underestimating the true intervention effect and increasing the risk of type II error.

To mitigate this risk, we have implemented several strategies. First, to reduce cross-arm communication among students, we instructed homeroom teachers to frame all intervention activities under the common label of “Health Education Courses” rather than as a research-specific program. Students are not informed of the existence of different intervention modalities, nor are they told which modality their class belongs to. This reduces the likelihood of students actively comparing and sharing activity content across classes. Second, intervention materials distributed to offline and combined classes (eg, pamphlets and mini-program links) are explicitly marked with the class name and are not disseminated to control classes. Teachers are reminded to avoid sharing activity content outside their assigned class. Third, the WeChat mini-program used for online activities is access controlled by class; control group students cannot log into the intervention platform. Fourth, as part of the process evaluation, we will monitor potential contamination through semistructured interviews by asking students in the control group whether they have heard about or seen any anti-smoking activities or materials from other classes.

In the statistical analysis, we will adopt a conservative analytical strategy. Under the intention-to-treat principle, all participants will be analyzed according to their original assigned groups regardless of actual exposure. If contamination occurs, it will only attenuate the observed effect size relative to the true effect; therefore, any statistically significant effect detected despite contamination would provide strong evidence of intervention efficacy. Additionally, in exploratory sensitivity analyses, we will exclude control group participants who report having been exposed to intervention content during the process evaluation interviews to assess the extent of contamination and its potential impact on the primary findings.

It is important to acknowledge that, because of this contamination risk, our trial design may yield conservative effect estimates. However, this conservative bias is acceptable for a pilot trial, as it ensures that any positive signal observed is robust and not merely an artifact of ideal but unrealistic implementation conditions. Should the intervention prove effective under these conservative conditions, it would provide strong support for future scale-up. Conversely, if contamination is found to be substantial, we will consider using a school-level randomization design (rather than class level) or larger separation margins between intervention and control classes in the definitive trial.

### Intervention Details

Once the students were enrolled, the classes were randomly assigned to receive either the anti-smoking intervention or the usual intervention. These intervention conditions were described as follows.

This school-family collaborative, online-offline integrated intervention is grounded in established behavior change techniques, and its design incorporates multiple key evidence-based components. First, the program adopts group support, professional guidance, and self-help materials—core intervention strategies widely recognized as effective in the field of adolescent health promotion [23]. Second, it leverages the well-established efficacy of mHealth tools in promoting adolescent health, including smoking cessation [24]. Finally, the program integrates a novel mobile app specifically developed for this study, whose design was directly informed by the empirically validated Smokerface app—an intervention that demonstrates the harms of smoking by simulating its aging effects and has been shown in randomized controlled trials to reduce smoking rates among adolescents [25]. This program was developed by a multi-institutional research team. The team members included mentors and graduate students from the Chinese Center for Disease Control and Prevention, Capital Medical University, and Beijing Center for Disease Control and Prevention. Once consensus was reached on the key messages, a series of anti-smoking educational materials were developed, and the training methods were revised based on suggestions from students, teachers, school physicians, and school administrators during formative evaluations. In each school, it was expected that at least two staff members (1 school physician and 1 district Centers for Disease Control and Prevention [CDC] staff member) received training. All participating students in the intervention group received anti-smoking knowledge from the school physician and district CDC staff. The school physician and district CDC staff received training before implementing the intervention. Attendance registers and staff supervision checklists were used for each offline activity. Score records were kept for knowledge competitions. For online knowledge quizzes and digital mini-games, backend statistics from WeChat were collected. The training lasted approximately 2 hours and covered finalization of intervention activity content, how to use the WeChat mini-program, and confirmation of intervention activity schedules. The training was delivered using a combination of offline and online meetings. Training effectiveness was assessed through hands-on demonstration by the trainees. The intervention content included the following:

- Three 30-minute lectures were delivered by district CDC staff over 2 semesters, covering the harms of tobacco, e-cigarettes, and SHS or thirdhand smoke. The intervention was provided to both the offline intervention classes and the hybrid (online and offline) intervention classes.
- Thirteen antismoking pamphlets were distributed in paper and electronic formats and shared in parent group chats. Students took them home, reviewed them with parents, and answered questions in an activity passport for joint engagement. The intervention was provided to all classes receiving the intervention.
- Five microvideos from the World Health Organization (WHO) were shared via parent WeChat groups, aimed at students and parents watching together. The intervention was provided to both the online intervention classes and the hybrid (online and offline) intervention classes.
- Two peer games were conducted: a card game on the health hazards of smoking and a role-playing game to practice refusing the first cigarette. The intervention was provided to both the offline intervention classes and the hybrid (online and offline) intervention classes.
- Four knowledge competitions were conducted by school staff to reinforce key messages from the lectures, pamphlets, and videos. The intervention was provided to both the offline intervention classes and the hybrid (online and offline) intervention classes.
- Two rounds of online knowledge quizzes were held. A WeChat mini-program for knowledge competitions was distributed via parent WeChat groups, using the shared item bank with in-person competitions. The intervention was provided to both the online intervention classes and the hybrid (online and offline) intervention classes.
- Two rounds of digital mini-games were conducted. The “Aging Appearance” WeChat mini-program was distributed via parent groups, enabling students and parents to visually observe smoking’s association with facial aging. The intervention was provided to both the online intervention classes and the hybrid (online and offline) intervention classes.

### Treatment as Usual

The control group received standard interventions, such as posting anti-smoking pamphlets at the school.

To ensure consistent delivery of the multicomponent intervention across schools, classes, and implementers, we conducted a standardized training session for all implementers prior to implementation. The training covered, for each of the 7 intervention components: (1) the core content and key messages delivered (eg, for lectures: the health harms of tobacco, e-cigarette constituents, and SHS or thirdhand smoke exposure); (2) the delivery format and duration (eg, 30-min lectures and 15-min peer games); (3) the target audience (students only vs students with parents); (4) the required materials and equipment (eg, lecture slides, pamphlets, and WeChat mini-program links); and (5) the scheduled timeline for each activity. All lecture slides, knowledge competition question banks, and activity passports were centrally developed by the multi-institutional research team and reviewed by tobacco control experts to ensure content accuracy and consistency. Pamphlets and microvideos were selected from WHO-recommended materials and pretested for age appropriateness with a small group of seventh-grade students during the formative evaluation phase. For the WeChat mini-program, a standardized user guide was provided to all participating parents and students, outlining step-by-step instructions for accessing online quizzes and the “Aging Appearance” digital game. By standardizing these core materials and delivery protocols, we aimed to minimize variability in intervention content across implementers and sites while allowing for minor contextual adaptations (eg, scheduling adjustments to accommodate school timetables) as documented in the process evaluation.

Intervention delivery was carried out by two types of implementers at each school: (1) school physicians (or health teachers), who coordinated on-site activities and facilitated school-level logistics; and (2) district CDC staff, who delivered lectures and supervised intervention activities. Prior to the start of the intervention, all implementers attended a centralized training session lasting approximately 2 hours. The training covered three modules: (1) an overview of the intervention rationale, components, and expected outcomes; (2) a detailed walkthrough of the standardized intervention manual, including the content and schedule of each activity; and (3) hands-on practice with the WeChat mini-program, including how to access the platform, monitor student participation via backend statistics, and troubleshoot common technical issues. The training was delivered through a combination of in-person and online meetings to accommodate implementers from the 3 geographically dispersed districts. Training effectiveness was assessed through a hands-on demonstration in which each trainee was required to successfully navigate the mini-program and deliver a 5-minute mock lecture segment, evaluated by the research team using a structured checklist. Refresher training and technical support were provided via a dedicated WeChat group throughout the intervention period, allowing implementers to ask questions and share experiences in real time.

Participants were free to withdraw from the intervention activities at any time without providing a reason and without any negative impact on their schooling or grades. Given the fixed educational nature of the intervention, no dose modifications or stepwise changes were applicable. If a student withdrew, they were still encouraged to complete the follow-up assessments unless they explicitly declined.

### Outcomes Assessment

#### Overview

The specific outcomes of this pilot trial include (1) the number of clusters (classes) and the size of each cluster (students) for the main trial; (2) recruitment and attrition rates for clusters and participants; (3) descriptive data on the characteristics of participating clusters and participants; (4) reasons for nonparticipation or nonconsent of clusters and participants; (5) estimate of the effect size of the primary outcome in the definitive trial (students’ knowledge, attitudes, and behavior level); (6) the costs associated with delivering the anti-smoking intervention; and (7) fidelity of the intervention.

The PRISM framework guided the evaluation of the implementation process at the levels of patients, providers, and the health system (Figure 2). Using a mixed methods approach, we will assess the following implementation outcomes: knowledge awareness of tobacco, attitudes toward smoking, current smoking prevalence, ever smoking prevalence, quit attempts among smoking family members and students’ refusal intentions, and parental-related smoking situations [26].

**Figure 2.**
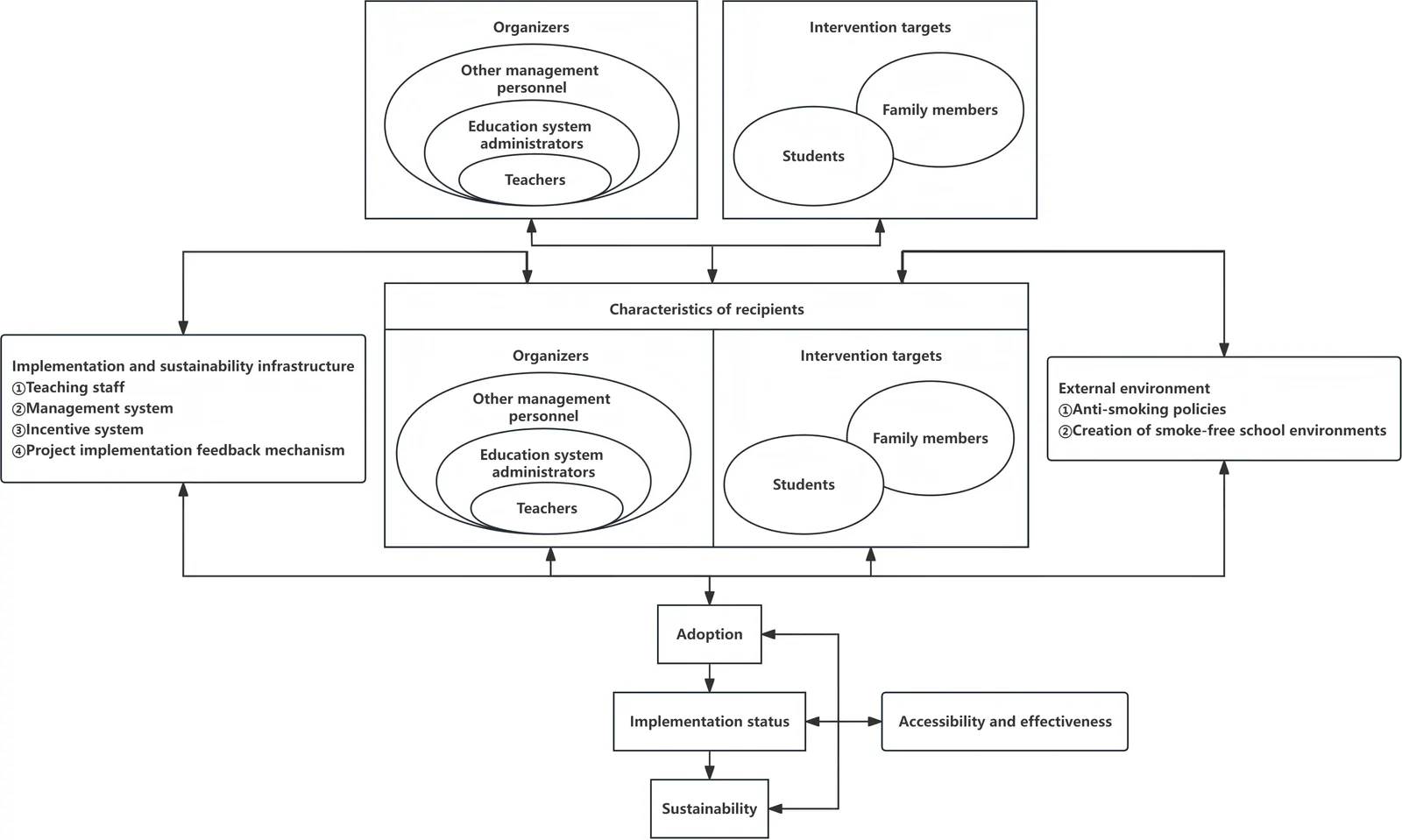
The PRISM (Practical, Robust Implementation and Sustainability Model) theoretical framework for the formation of tobacco control intervention model.

We will assess and report on these outcomes as part of our data collection and analysis plan. The outcomes for the definitive trial were measured before and after the intervention in each of the study’s arms. We first describe these outcomes and then the process and schedule of assessing these (Table 1).

Consistent with the pilot nature of this trial, this study has coprimary outcome domains: implementation process and feasibility indicators (fidelity, participation, satisfaction, and contextual factors) as guided by the PRISM framework; and changes in tobacco-related knowledge and attitudes, which represent the proximal cognitive targets of the intervention. Tobacco-related behaviors (smoking prevalence, cessation attempts, and household SHS exposure) are specified as secondary outcomes, given their low baseline prevalence in this age group and the short follow-up duration, making them more suitable for hypothesis generation than confirmatory testing.

**Table 1.** Summary of outcome measures and follow-up.

Outcomes	Outcome measure	Baseline	3 months	6 months
Primary: process, feasibility, and cognitive outcomes				
Fidelity	Proportion of intervention activities delivered as planned (derived from activity logs and staff checklists)		✓	✓
Participation	Student attendance rates for offline activities; completion rates for online activities (backend statistics from WeChat mini-program)		✓	✓
Satisfaction and contextual factors	Semistructured interview data capturing participants’ and providers’ satisfaction with intervention content, format, and frequency; barriers and facilitators identified through thematic analysis (PRISM^a^ framework)		✓	
Tobacco-related knowledge	Composite score of secondhand smoke-related disease cognition; correct judgment on thirdhand smoke harmfulness; correct judgment on e-cigarette addictiveness; correct judgment on whether e-cigarette aerosol contains harmful substances and nicotine	✓		✓
Tobacco-related attitudes	Proportion of students who perceive smoking as attractive; proportion who believe smoking facilitates social comfort	✓		✓
Secondary: behavioral outcomes				
Tobacco-related behaviors	30‑day smoking prevalence; ever smoking prevalence; future smoking intention; quit attempts (among smoking family members) and students’ refusal intentions or resistance to peer pressure	✓		✓
Household smoke exposure attitudes	Proportion of parents smoking at home; proportion smoking in children’s presence; days of indoor smoking in the past week; thirdhand smoke exposure (smell on household furniture or clothing in the past week)	✓		✓

a PRISM: Practical, Robust Implementation and Sustainability Model.

#### Primary Outcome

The primary outcomes are as follows:

1. Implementation and feasibility outcome: Whether the intervention is implemented as planned; participants’ suggestions regarding intervention modality, content, and frequency; and the participation, adherence, and satisfaction of both recipients and implementing organizations. The specific criterion is the authentic statements expressed by interviewees during the qualitative interviews.
2. Cognitive outcome: Changes in the level of tobacco-related health knowledge and attitudes. Specific indicators include a composite score of knowledge regarding diseases caused by SHS, correct judgment on the harmfulness of thirdhand smoke, correct judgment on the addictiveness of e-cigarettes, correct judgment on whether e-cigarette aerosol contains harmful substances, correct judgment on whether e-cigarettes contain nicotine, the proportion of students who perceive smoking as attractive, and the proportion who believe smoking facilitates social comfort. Generalized estimating equations (GEEs) will be used to assess the differential changes from baseline to endline between the intervention groups and the control group on the above outcome indicators (ie, the time×intervention interaction term), reflecting the net effect of the intervention.

#### Secondary Outcomes

Changes in students’ tobacco-related behaviors will be assessed. Specific indicators include students’ future smoking intentions, 30-day smoking prevalence, ever smoking prevalence, the proportion who have discouraged others from smoking, parental smoking rates, quit attempts, cigarette consumption, the proportion of parents who smoke at home, the proportion who smoke in the presence of their children, and thirdhand smoke exposure (smell on household furniture or clothing in the past week).

Other indicators include sociodemographic characteristics.

### Data Collection

Prior to randomization, a baseline assessment included a questionnaire. The questionnaire comprised a self-designed basic information sheet and a tobacco control questionnaire developed through expert discussion. The questionnaire includes the Chinese Version of the Ten-Item Personality Inventory and other conventional tobacco control survey items, with a total of 54 items. Missing data were handled using imputation methods. The final assessment took place at month 7 post-allocation, and all assessments conducted at baseline were repeated at the follow-up.

The full baseline and endline questionnaires are provided in Multimedia Appendices 1 and 2. The questionnaire covers sociodemographic, tobacco use, SHS and thirdhand smoke exposure, e-cigarette use, tobacco-related knowledge and attitudes, the Chinese version of the Ten-Item Personality Inventory (TIPI-C), and attitudes toward intervention activities. All items were developed based on the PRISM dimensions and expert review. The final questionnaire was further pilot-tested with a small sample of 40 seventh-grade students (not participating in the main trial) to assess comprehension and face validity, leading to minor wording adjustments for improved clarity. While we acknowledge that formal psychometric properties (eg, test-retest reliability and construct validity) have not yet been established, given the pilot nature of this trial and the context-specific adaptation of some items, the expert consensus and pilot testing procedures provide initial content validity.

We selected one school from each district (3 schools in total, with 3 districts) to participate in the interviews. A portion of students, teachers, grade leaders, school physicians, and district CDC staff from these 3 schools (12 students, 1 class teacher, 1 grade leader, 1 school physician, and 1 CDC staff member from each school) were interviewed 2 months after the tobacco control intervention was implemented. The semistructured interviews explored the barriers to and facilitators of implementing the different components of the tobacco control intervention, including training and resource needs. The interviews were conducted face-to-face at the schools. If face-to-face interviews were not possible, they were conducted via Tencent Meeting. With the participants’ consent, the interviews were digitally recorded and transcribed verbatim, with all personal data anonymized.

### Data Management and Archiving

The data were initially collected in the form of paper-based questionnaires. These questionnaires, which do not contain any identifiable information about the participants, were collected by the research team and stored in a locked cupboard separate from the consent forms. Data were entered into the trial database weekly, and the database was kept on a secure server. The paper copies continued to be stored in a securely locked cupboard. All data generated by this study will be securely stored by China CDC and Capital Medical University for a minimum of 5 years (calculated from the end of the study). The storage period is set in accordance with the Personal Information Protection Law of the People’s Republic of China, the Data Security Law, and the requirements of the China CDC and Capital Medical University Ethics Committee, ensuring data traceability and meeting potential audit needs.

A formal Data Monitoring Committee was not established for this pilot trial, as the intervention is a low-risk educational program with no drugs, devices, or invasive procedures, and no anticipated serious adverse events; however, we have a corresponding data management plan. Trial conduct, participant safety, and data integrity were monitored directly by the principal investigators.

### Statistical Considerations

#### Sample Size

This study is designed as a pilot cluster randomized controlled trial with a dual purpose to evaluate the feasibility and acceptability of the multicomponent intervention and its implementation processes (including fidelity, participation, and satisfaction), and to provide preliminary estimates of intervention effects on tobacco-related knowledge, attitudes, and behaviors to inform sample size planning for a future definitive trial. Given this dual-purpose design, the sample size calculation was primarily based on the primary effectiveness outcome—awareness of tobacco-related diseases—to ensure sufficient precision for effect estimation, while acknowledging that the trial is not powered to detect small differences in behavioral outcomes.

The sample size was calculated based on the primary effectiveness outcome (awareness of tobacco-related diseases) using a formula for multiple group comparisons. Assuming a cluster size of 40, a 2-sided α of .05, power of 0.90, and an intracluster correlation coefficient of 0.02, the design effect was estimated. To ensure sufficient precision for the pilot trial and to account for potential subgroup exploration, we inflated the sample to 1280 participants (320 per group, 8 classes per group) [27-29]. Detailed calculation formulas are provided in Multimedia Appendix 3.

To ensure adequate statistical power and provide sufficient precision for exploratory subgroup analyses, we doubled the required sample size to a total of 1280 participants (320 per group, corresponding to 8 classes per group). The use of class-level (rather than individual-level) randomization accounts for the natural clustering of students within classrooms and aligns with the real-world school structure, where health education is typically delivered at the class level. This design also minimizes the risk of within-class cross-contamination that would occur if individual students within the same class were randomized to different arms.

As this is a pilot trial, all effectiveness outcomes (eg, changes in tobacco-related knowledge, attitudes, and behaviors) are considered exploratory and will be interpreted with appropriate caution. Feasibility and implementation outcomes—including fidelity, participation, and satisfaction—constitute the primary focus of this study.

#### Statistical Analysis

The primary analysis strategy is intention-to-treat, in which all participants who completed the baseline survey and underwent randomization are included in the statistical analysis according to their original assigned groups, regardless of their actual degree of intervention exposure.

Missing data are anticipated in both baseline and endline questionnaires due to student absenteeism or incomplete responses. To handle missingness while preserving statistical power and minimizing bias, we will adopt a multiple imputation approach under the missing at random assumption. Specifically, we will generate 20 imputed datasets using chained equations, incorporating all variables in the analytical model—including outcome variables (knowledge scores, attitude indicators, and behavioral measures), covariates (gender, district, living arrangement, and personality traits), and auxiliary variables (class-level attendance rates and intervention arm indicators)—to improve imputation precision [30]. The GEE analysis will be performed on each imputed dataset, and the results will be pooled using Rubin’s rules [31] to obtain combined parameter estimates and standard errors that appropriately reflect both within-imputation and between-imputation variability.

The process evaluation is a qualitative study. Phenomenology is the most commonly used research method in qualitative research, among which the Colaizzi 7-step method is the most frequently adopted data analysis approach within descriptive phenomenological research [32]. Data extraction and analysis will be conducted using NVivo 12.0 (Qualitative Solution and Research International) in conjunction with the Colaizzi 7-step method: (1) familiarization with the data—reading and re-reading all transcripts to acquire an overall sense of the content; (2) identification of significant statements—extracting phrases and sentences directly relevant to the phenomenon under investigation; (3) formulation of meanings—interpreting the meaning of each significant statement; (4) clustering of themes—organizing the formulated meanings into theme clusters and categories; (5) developing an exhaustive description—integrating all themes into a comprehensive account of the experience; (6) producing the fundamental structure—distilling the exhaustive description into a concise and fundamental structural framework; and (7) returning to participants for validation—presenting the findings to a subset of interviewees to verify the accuracy and completeness of the interpretation.

For outcome indicators, GEE corrects for within-cluster correlation by specifying a plausible working correlation matrix (exchangeable structure is adopted in this study), thereby yielding more robust parameter estimates. In this model, the interaction term “intervention mode×time” is included to estimate and compare the effect sizes (regression coefficient β or odds ratio) of each mode relative to the control group. By examining the β (or odds ratios), 95% CIs, and *P* values corresponding to each mode, the relative efficacy of different intervention pathways can be scientifically determined.

Consistent with the pilot nature of this trial, all effectiveness outcomes, particularly behavioral measures such as smoking prevalence and cessation—are considered exploratory and hypothesis-generating. The primary focus of the statistical analysis is on estimating feasibility, implementation fidelity, and changes in cognitive outcomes (knowledge and attitudes), which will inform sample size calculations and design choices for a future definitive trial. To compare the differences in tobacco control effects among the intervention modes (online, offline, and combined online-offline) relative to the control group, while controlling for the correlation of repeated measures within individuals, this study adopts GEE for modeling analysis. GEE is suitable for longitudinal data or outcome variables with intracluster correlation in cluster randomized controlled trials, and it obtains robust parameter estimates by specifying a working correlation matrix to adjust for within-cluster correlation.

To estimate intervention effects while accounting for within-cluster correlation, we will use GEEs with an exchangeable working correlation matrix. The primary parameter of interest is the time-by-group interaction term, which represents the net effect of each intervention arm relative to the control. The GEE models will include covariates such as gender, district, living arrangement, and personality traits. Detailed model specifications, including the link functions and equation forms, are provided in Multimedia Appendix 3.

Considering the potential correlation between the 2 repeated measurements for the same individual, an exchangeable working correlation matrix is specified, and robust SEs are used to obtain consistent inferences for parameter estimates. Even if the working correlation matrix is misspecified, this approach still provides valid statistical tests. All analyses are based on GEE models, systematically comparing the relative efficacy of different intervention modes by estimating the β and its 95% CIs and *P* values for each intervention group.

Given that testing multiple related knowledge-attitude-behavior outcome variables simultaneously increases the risk of type I error, the *P* values derived from GEE analyses will be adjusted using the false discovery rate (FDR) correction method. The FDR correction controls the expected proportion of false rejections (ie, false positives) among multiple hypothesis tests. Compared with traditional methods such as Bonferroni, it has greater statistical power and effectively reduces false negatives resulting from overcorrection. The specific correction procedure involves, first, sorting the raw *P* values from all tests in ascending order as *P*₁≤*P*₂≤...≤*P*_m_, setting the FDR level at an α of .05 and then identifying the largest k satisfying $P_{k}\leq \left(\frac{k}{m}\right)a$, and rejecting the null hypotheses corresponding to *P*₁...P_k_. In reporting, both raw *P* values and FDR-adjusted *P* values will be presented to enhance the robustness of statistical inference and balance the risks of false positives and false negatives.

To explore whether intervention effects are heterogeneous across individual or environmental characteristics, we will further conduct subgroup analyses. The overall sample will be stratified into subgroups based on variables such as gender, region of residence, personality traits, and family co-habitation status. GEE will be independently run within each subgroup, and by comparing the magnitude, direction, and significance levels of intervention effects (β) across subgroups, we can identify which subpopulations benefit more from the intervention.

For stratified analyses, we will perform gender-stratified analyses and region-stratified analyses.

#### Implementation Evaluation

Drawing on the 4 core dimensions of PRISM, the intervention, the recipients, the external environment, and the implementation and sustainability infrastructure, we operationalized each dimension into measurable constructs for this pilot trial. The PRISM framework derived constructs, their operational definitions, data sources, and the levels at which they are assessed (recipient, provider, or health system). These constructs guided the development of our semistructured interview guides, the selection of quantitative implementation indicators (fidelity, participation, and satisfaction), and the interpretation of both process and outcome findings. By systematically mapping our evaluation measures to PRISM dimensions, we aim to move beyond simply assessing “whether the intervention worked” to understanding “why it worked (or did not)” and “under what contextual conditions.” We used the PRISM framework to evaluate the implementation of the multicomponent program [33]. The implementation evaluation allowed us to monitor and improve program implementation, understand the relationship between implementation characteristics and health outcomes, and design the dissemination plan if the program is proven effective. We assessed the PRISM dimensions at the recipient, provider, and health system levels (refer to Multimedia Appendix 4). We used a combination of quantitative and qualitative methods to assess the domains of interest. We collected data during patients’ study visits at 2 months and 7 months. Additionally, we regularly (every 1‐2 weeks) contacted district CDC staff and school medical staff via WeChat to capture study inputs and ongoing program implementation, recording these through checklists. At some study schools, we conducted semistructured interviews and direct observations with participants, providers, and public health administrators. Fidelity and satisfaction were primarily determined based on the content of the interviews; no specific predefined scoring criteria were established.

#### Qualitative Evaluation

We conducted semistructured interviews to explore participants’ and implementers’ experiences, perceptions, and suggestions regarding the intervention. Interviews were carried out approximately 2 months after the intervention started (March 2025) and involved students, homeroom teachers, grade leaders, school physicians, and district CDC staff. Purposive sampling was used to select one school from each district (Fengtai, Tongzhou, and Fangshan), and within each school, 12 students (4 from each intervention arm), 1 homeroom teacher, 1 grade leader, 1 school physician, and 1 CDC staff member were recruited, yielding a total of 48 interviews. The interview guides (provided in Multimedia Appendix 1) were structured around the four PRISM dimensions: (1) intervention (activity participation, frequency, and content suggestions); (2) recipients (satisfaction, engagement, adherence, and preferences); (3) external environment (school tobacco policy, community activities, and family smoking norms); and (4) implementation and sustainability infrastructure (training, supervision, and resource support). Separate versions were developed for students and organizational staff. Each interview lasted approximately 10 to 15 minutes, was conducted face-to-face in a private setting at school, and was audio-recorded with participants’ consent. Field notes on nonverbal cues and contextual factors were also taken.

All interview recordings were transcribed verbatim in Chinese. Transcripts were not returned to participants for correction, but member checking was conducted by summarizing emerging themes and presenting them to a subset of interviewees to verify interpretive accuracy. As mentioned, data analysis followed the Colaizzi 7-step phenomenological analysis method, which is the most commonly used approach within descriptive phenomenological research.

To ensure rigor, we adopted several trustworthiness strategies aligned with Lincoln and Guba’s criteria [34]. Credibility was enhanced through persistent observation, investigator triangulation (2 researchers independently code a subset of transcripts and resolve discrepancies through discussion), and member checking as described earlier. Transferability was supported by thick description of the study context, participant characteristics, and the intervention setting, enabling readers to assess applicability to their own contexts. Dependability was established by maintaining an audit trail that documents all analytical decisions, coding revisions, and theme development, with regular debriefing sessions among the research team. Confirmability was ensured by explicitly acknowledging the researchers’ preexisting perspectives and by using reflective memos throughout the analysis to bracket assumptions. Reflexivity was a central component: the lead qualitative analyst kept a reflexive journal, and team discussions critically examined how the researchers’ backgrounds (as public health professionals and tobacco control researchers) may shape interpretation. We also actively searched for disconfirming evidence (negative cases) to challenge and refine emerging themes.

Data saturation was assessed by reserving 2 student and 2 staff interviews as a hold-out sample; after initial thematic structure was developed, these reserved transcripts were coded independently. If no new themes or substantial modifications emerged, saturation was considered reached.

### Trial Registration

This trial was registered with the Chinese Clinical Trial Registry under registration number ChiCTR2500108279. The registration was completed after the initiation of participant recruitment (retrospective registration). The delay in registration was due to administrative timelines in the trial preparation phase; the study protocol had been finalized, and ethics approval had been obtained prior to recruitment, and no changes were made to the primary outcomes, intervention components, or analytical plan between the start of the study and registration. The authors confirm that all outcomes specified in the registered protocol are reported in this manuscript. Full details of the trial registry entry are available at [35].

### Ethical Considerations

This study was conducted in full compliance with the Declaration of Helsinki (1996 version) and the relevant Chinese regulations on the protection of human subjects in biomedical research.

Ethical approval has been obtained from the Ethics Committee of Capital Medical University (approval Z2024SY078) and the Ethics Review Committee of the Chinese Center for Disease Control and Prevention (approval 202432). Any protocol amendments during the trial will be submitted for re-approval to both committees before implementation.

Written informed consent was obtained from the parents or legal guardians of all participating students prior to enrollment. The consent form clearly explained the study purpose, procedures, potential risks and benefits, and the voluntary nature of participation. In addition, verbal assent was obtained from each student participant after age-appropriate information about the study had been provided. Participants were explicitly informed that they could withdraw from the study at any time without providing a reason and that their decision would not affect their schooling, grades, or any other services they are entitled to receive.

Strict measures were implemented to protect participants’ privacy and data confidentiality. All paper-based questionnaires were anonymized and deidentified immediately upon collection; they were stored in a locked filing cabinet that is separate from signed consent forms. Digital data, including audio-recorded interview files and transcribed texts, were stripped of any personal identifiers (eg, names and school names) and stored on a password-protected secure server with access restricted to the core research team. Data will be retained for a minimum of 5 years after study completion, in accordance with Chinese data protection laws (Personal Information Protection Law and Data Security Law), and will be securely destroyed thereafter.

Participants did not receive monetary compensation for their involvement. However, to acknowledge their time and contribution, all students who completed the full intervention activities and follow-up assessments received small nonmonetary incentives, such as participation certificates and modest study-related stationery items (eg, notebooks and pens), as permitted by the ethics committees. No compensation was provided to schools or staff beyond the routine educational materials and activity supplies covered by the study budget.

## Results

Recruitment has been completed with 1301 participants enrolled. Minor deviations from the original protocol occurred during implementation, as detailed in the following subsection; these did not compromise the overall integrity of the trial.

### Study Status and Participant Flow

Recruitment took place from July to November 2024. Of the 1410 students assessed for eligibility, 1330 met the inclusion criteria and were randomized by class into 4 arms: control (n=334), online intervention (n=336), offline intervention (n=335), and combined online-offline intervention (n=325). After excluding participants who declined to participate before the intervention (n=80) and invalid questionnaires at follow-up (intervention group: n=27; control: n=5), plus 2 losses to follow-up in the control group, a total of 1301 students (intervention: n=974; control: n=327) were included in the final analysis. The achieved sample size exceeded the prespecified target of 1280, providing adequate statistical power.

### Baseline Characteristics

The 4 groups were generally comparable in sociodemographic characteristics, including sex, area of residence, and most personality trait dimensions. The majority of students lived with both parents (82.5%‐88.7% across groups). Small baseline imbalances were observed for living arrangement and some personality traits (eg, agreeableness, openness), as well as for baseline SHS-related knowledge scores and thirdhand smoke exposure. These differences were addressed by including the corresponding covariates in the GEE models for outcome evaluation (Multimedia Appendix 5).

### Deviations From the Original Protocol

The intervention was implemented largely as planned across the 7-month period (November 2024 to June 2025). However, process evaluation interviews revealed minor deviations: in some schools, online-only and offline-only classes inadvertently received mixed activities (eg, offline group students reported watching microvideos, and online group students attended lectures), indicating some blurring of delivery boundaries. Additionally, 1 class that showed low cooperation from the homeroom teacher was replaced with another class from the same school to maintain intervention fidelity. Parental engagement varied considerably, with approximately one-third of parents showing active participation, while others declined or did not respond, which is a common challenge in school-based health programs.

### Timeline of Data Collection

Baseline survey was completed in November 2024. The intervention was delivered from November 2024 to June 2025, with a midpoint process evaluation (semistructured interviews) conducted in March 2025. Endline survey was finished in June 2025. Data cleaning and coding were carried out between July and August 2025. Quantitative and qualitative analyses commenced in September 2025, and the final results are expected to be reported in 2026.

## Discussion

### Anticipated Outcomes

On the basis of the completed implementation and preliminary observations, this protocol is expected to generate valuable findings across 3 levels.

At the cognitive level, the intervention is anticipated to significantly enhance adolescents’ knowledge of tobacco hazards, particularly regarding the disease spectrum caused by SHS, the residual harms of thirdhand smoke, and the addictiveness and chemical composition of e-cigarettes. Different delivery modes may exert their effects on distinct cognitive targets. The online-only arm, leveraging the convenience of the WeChat platform and the shared family-viewing context, is expected to perform better on knowledge breadth, such as comprehensive SHS-related disease cognition. In contrast, the offline arm, through face-to-face lectures, peer-led games, and knowledge competitions, is expected to be more effective in correcting specific misconceptions, such as “thirdhand smoke is harmless” or “e-cigarettes are not addictive.” The combined online-offline arm may harness the strengths of both modalities, potentially producing a synergistic effect, particularly in conveying fine-grained information (eg, e-cigarettes contain nicotine). Consistent with our predefined outcome hierarchy, these cognitive changes constitute the coprimary effectiveness end points, while behavioral shifts (eg, reduced household smoking) will be treated as secondary, hypothesis-generating outcomes due to the limited follow-up duration and low baseline behavioral prevalence.

At the behavioral and environmental level, the protocol is expected to observe reductions in household secondhand and thirdhand smoke exposure. Preliminary process evaluation data suggest that some parents with smoking habits, after participating in shared activities, such as reading leaflets, watching microvideos, and playing the “aging appearance” game together with their children, demonstrated a willingness to reduce or quit smoking. This “spillover effect” aligns with the findings of Sánchez-Franco et al [36] in their cross-country comparative study: adolescents can act as “health catalysts,” transmitting knowledge and beliefs acquired from interventions back to family members. However, it must be acknowledged that, given the extremely low baseline smoking rate among the participating middle school students (below 2%), changes in students’ own smoking behaviors are unlikely to be detected within the short follow-up period. This observation underscores the need for extended follow-up durations or targeted subgroup analyses focused on high-risk populations in future intervention designs.

At the implementation level, the process evaluation guided by the PRISM framework is expected to reveal several generic implementation barriers, including poor school-family communication leading to low parental engagement, weak community tobacco control resources that fail to provide environmental support, and blurred boundaries between online and offline activities in practice. Conversely, facilitating factors are also anticipated, such as the strong student appeal of incentive-driven activities (knowledge competitions and peer games) and the effectiveness of homeroom teacher supervision and student assistant training in enhancing participation adherence. These findings will provide actionable evidence for iterative optimization and cross-regional scale-up of the intervention.

### Comparison and Innovation Over Existing Interventions

Comparing the design logic of this protocol with representative existing studies further highlights its rationality and innovation.

In the domain of school-based tobacco control, the X:IT cluster randomized trial conducted by Bast et al [37,38] in Denmark bears the closest resemblance to our protocol. That study also used the class as the unit of randomization and adopted a multicomponent design (school policy, parental involvement, and peer interaction). However, the X:IT study encountered low adherence in the parental involvement component, with many parents citing “lack of time” or “perceiving it as unnecessary,” highlighting the need for more refined school-family linkage mechanisms. Our protocol reduces the participation threshold for parents through regular homeroom teacher reminders and WeChat-based material distribution, allowing engagement via mobile devices without requiring physical attendance. We include online-only and combined arms to accommodate parents’ fragmented schedules. Preliminary process feedback indicates that most parents held positive attitudes toward the activities, although their willingness to participate still showed polarization.

In terms of digital interventions, the Click City program developed by Andrews et al [39] in the United States is a fully digital, school-based curriculum that achieved an 85% completion rate, demonstrating the feasibility of digital delivery in school settings. However, Click City’s limitation lies in its lack of family-level reach. Our protocol, in contrast, directly delivers intervention components to the home setting via WeChat miniprograms and parent groups, enabling parent-child co-learning and co-play. This design is particularly well-suited to the Chinese context, given the near-universal penetration of WeChat among parents of primary and secondary school students, and the cultural emphasis on shared parent-child educational activities. It is expected to expand intervention coverage and depth without unduly increasing the administrative burden on schools.

The Education Against Tobacco program, evaluated by Brinker et al [25,40] in German secondary schools, used a “face-aging” mobile app to visually demonstrate the effects of smoking on skin aging, a representative mHealth intervention. However, the randomized controlled trial showed no significant reduction in smoking rates, with a dropout rate as high as 35%. The researchers attributed this to the lack of sustained behavioral guidance mechanisms beyond the initial “novelty effect” of the app. Our protocol positions the “face-aging” game as only 1 of 7 components in the online arm, complemented by knowledge competitions, peer role-playing, and parent-child discussions to sustain engagement. Furthermore, institutionalized participation reminders through homeroom teachers and school physicians are expected to substantially reduce attrition.

Regarding contextual adaptability, Sánchez-Franco et al [36] through a qualitative comparative study between the United Kingdom and Colombia, found that the effectiveness of adolescent smoking prevention interventions is highly dependent on regional-level factors, including community tobacco control resources, policy enforcement intensity, and social norms. This finding strongly supports the design logic of our protocol, which covers 3 distinct development tiers: Fengtai (urban core), Tongzhou (peri-urban), and Fangshan (suburban). Baseline data are expected to reveal variations across these regions in household smoking rates, community activity frequency, and school tobacco control climates, which may further moderate the magnitude and direction of intervention effects. If confirmed, this finding will provide direct evidence for implementing regionally tailored, precision tobacco control strategies.

Taken together, by integrating “school-family dual agency, online-offline dual channels, and PRISM four-dimensional evaluation,” our protocol retains the effective components of prior studies while systematically addressing their common shortcomings, insufficient parental engagement, effect decay, and weak contextual adaptation. This design is expected to yield more stable and generalizable implementation outcomes.

### Strengths and Limitations

The strengths of the protocol are fourfold. First, the cluster randomized design closely mirrors the real-world operational context of schools. Randomizing by class rather than by individual student avoids cross-contamination between different intervention modalities within the same class and reduces administrative complexity, thereby enhancing the generalizability of the intervention. Second, the 3-tier regional stratification (urban, peri-urban, and suburban) covers a gradient of development levels, increasing the external validity and contextual applicability of the conclusions. Third, the mixed methods design (quantitative outcome evaluation plus qualitative process evaluation) addresses both the “what” and the “why,” meeting the methodological requirements of implementation science for mechanistic explanation. Fourth, the head-to-head comparison of 3 delivery modes provides clear, evidence-based guidance for resource-constrained settings (where simultaneous implementation of both online and offline activities may not be feasible), offering direct decision-making utility.

The limitations of the protocol must also be candidly acknowledged. First, the follow-up period is limited to a single academic year (approximately 7 mo). While this duration is sufficient to capture medium-term cognitive changes, it cannot assess the actual incidence of smoking initiation or the long-term maintenance of cessation behaviors. Given that tobacco addiction typically peaks in late adolescence (after age 15 years) [41], the age window of our participants (12‐13 y) is better suited for “prevention-oriented cognition formation” than “behavioral correction.” Long-term behavioral outcomes would require extended follow-up into high school.

Second, the quantitative outcomes rely primarily on self-reported questionnaires. Although anonymity was ensured to minimize social desirability bias, sensitive items, such as smoking behavior and cessation advice, may still be subject to underreporting or overreporting. Future studies could consider incorporating objective biochemical markers, such as salivary cotinine testing, to validate self-reported data.

Third, cluster randomization at the class level may introduce baseline imbalances. Our baseline data showed statistically significant between-group differences (*P*<.05) in certain indicators, including living arrangements, SHS-related knowledge scores, and thirdhand smoke exposure. While the analysis plan includes covariate adjustment via GEEs, it must be acknowledged that cluster randomization cannot fully balance confounding factors to the same extent as individual-level randomization. Interpretation of results should therefore remain cautious.

Fourth, the online intervention relies on the WeChat platform and parents’ smartphone proficiency, which may create participation barriers for families with lower digital literacy and thus compromise equity. Process evaluation feedback already indicated that some parents “did not receive messages” or “could not operate the miniprogram,” suggesting the need for simple technical support in future scale-up.

Fifth, the process evaluation did not directly interview parents or school principals. Information regarding parental attitudes, changes in household smoking behavior, and school-level organizational support was obtained indirectly through students and front-line implementers (homeroom teachers and school physicians), which may be subject to information attenuation or reporting bias. Should conditions allow, future formal evaluations could supplement the process analysis with direct interviews of parents and school leadership.

## Supplementary material

10.2196/86503 Multimedia Appendix 1 Baseline questionnaire.

10.2196/86503 Multimedia Appendix 2 Interview guide.

10.2196/86503 Multimedia Appendix 3 Sample size and GEE model specifications.

10.2196/86503 Multimedia Appendix 4 Implementation evaluation.

10.2196/86503 Multimedia Appendix 5 Baseline data.

10.2196/86503 Checklist 1 SPIRIT 2025 checklist.

## Abbreviations

CDC: Centers for Disease Control and Prevention
FDR: false discovery rate
GAIDeT: Generative AI Delegation Taxonomy
GEE: generalized estimating equation
Gen-AI: generative AI
mHealth: mobile health
PRISM: Practical, Robust Implementation and Sustainability Model
RE-AIM: reach, effectiveness, adoption, implementation, and maintenance
SHS: secondhand smoke
TIPI-C: Chinese Version of the Ten-Item Personality Inventory
WHO: World Health Organization
