# Supplementary material for: School-Family Linked and Online-Offline Combined Tobacco Control Intervention for Adolescents: Protocol for a Cluster Randomized Pilot Trial
Source: JMIR Res Protoc. 2026 Oct 21;15:e86503. doi: 10.2196/86503 (PMC13686337; doi:10.2196/86503)
Supplement: Supplementary file 2 [file resprot-v15-e86503-s001.docx]

**2024 Beijing Grade 7 Student Smoking‑Related Survey Questionnaire**

_____ County (City/District) ______ School _____ Class Student ID ________

Hello! You have been selected to participate in the Beijing Grade 7 Student Smoking‑Related Survey. Your responses will be kept strictly confidential, including from your classmates, teachers, and parents. Your participation is very important for us to understand the real situation. There are no right or wrong answers to the questions. We will only use aggregated information and will not use your personal information. The survey results will be used as a reference for tobacco control activities. If you agree to participate in this survey, please fill in the questionnaire yourself; if you do not agree, you may return the blank questionnaire. Thank you for your cooperation and support!

I. General Information

1. Gender ( )
① Male ② Female

2. Age: ____ years old

3. Who do you live with at home? (Select all that apply)
① Father ② Mother ③ Grandfather (paternal) ④ Grandmother (paternal) ⑤ Grandmother (maternal) ⑥ Grandfather (maternal) ⑦ Other, please specify ______

4. How much pocket money do you have available each month? About ______ yuan.

5. Please select the option that best describes you according to your level of agreement with the following statements.

5.1. ( ) I see myself as outgoing and active.
① Strongly disagree ② Disagree ③ Slightly disagree ④ Neutral ⑤ Slightly agree
⑥ Agree ⑦ Strongly agree

5.2. ( ) I tend to be quiet and reserved.
① Strongly disagree ② Disagree ③ Slightly disagree ④ Neutral ⑤ Slightly agree
⑥ Agree ⑦ Strongly agree

5.3. ( ) I am usually caring and kind to others.
① Strongly disagree ② Disagree ③ Slightly disagree ④ Neutral ⑤ Slightly agree
⑥ Agree ⑦ Strongly agree

5.4. ( ) I prefer an easygoing and independent lifestyle and do not care much about others.
① Strongly disagree ② Disagree ③ Slightly disagree ④ Neutral ⑤ Slightly agree
⑥ Agree ⑦ Strongly agree

5.5. ( ) I always complete tasks as planned and take my responsibilities seriously.
① Strongly disagree ② Disagree ③ Slightly disagree ④ Neutral ⑤ Slightly agree
⑥ Agree ⑦ Strongly agree

5.6. ( ) I am often casual and do not pay much attention to details and plans.
① Strongly disagree ② Disagree ③ Slightly disagree ④ Neutral ⑤ Slightly agree
⑥ Agree ⑦ Strongly agree

5.7. ( ) I often feel anxious or uneasy.
① Strongly disagree ② Disagree ③ Slightly disagree ④ Neutral ⑤ Slightly agree
⑥ Agree ⑦ Strongly agree

5.8. ( ) I feel that I am emotionally stable and can handle stress.
① Strongly disagree ② Disagree ③ Slightly disagree ④ Neutral ⑤ Slightly agree
⑥ Agree ⑦ Strongly agree

5.9. ( ) I like to try new things and am interested in different cultures and ideas.
① Strongly disagree ② Disagree ③ Slightly disagree ④ Neutral ⑤ Slightly agree
⑥ Agree ⑦ Strongly agree

5.10. ( ) I prefer to keep things as they are and do not like change or novelty.
① Strongly disagree ② Disagree ③ Slightly disagree ④ Neutral ⑤ Slightly agree
⑥ Agree ⑦ Strongly agree

II. Cigarette Use

1. ( ) Have you ever tried smoking cigarettes, even just a puff or two?
① Yes ② No

2. ( ) In the past 30 days, on how many days did you smoke cigarettes?
① 0 days ② 1‑2 days ③ 3‑5 days ④ 6‑9 days ⑤ 10‑19 days ⑥ 20‑29 days ⑦ 30 days

3. ( ) In the past 30 days, on average, how many cigarettes did you smoke per day?
① 0 cigarettes ② Less than 1 cigarette ③ 2‑5 cigarettes ④ 6‑10 cigarettes ⑤ 11‑20 cigarettes ⑥ More than 20 cigarettes

4. ( ) Where do you usually smoke?
① I do not smoke ② At home ③ At a friend's home ④ At school ⑤ At an arcade/game hall ⑥ Other, please specify ______

5. ( ) Do you think you will smoke in the next 12 months?
① Yes ② No

6. ( ) How much money do you spend on buying cigarettes per month on average?
① I do not smoke cigarettes ② Please fill in ______ yuan

Ⅲ. Quitting Smoking

1.( ) Do you want to quit smoking now?
① I do not smoke ② Yes ③ No

2.( ) In the past 12 months, have you tried to quit smoking?
① I have never smoked ② I used to smoke but have not smoked in the past 12 months ③ Yes ④ No

3.( ) Have you ever received help or advice to quit smoking?
① I have never smoked ② Yes, from an organized quit‑smoking activity or professional ③ Yes, from friends ④ Yes, from family members ⑤ Yes, from both organized/professional and friends or family ⑥ I have not received any help to quit smoking

IV. Secondhand Smoke Exposure

1. ( ) How do you feel when you smell cigarette smoke?
① Like it ③ Indifferent ② Dislike it

2. ( ) How do you feel when someone smokes in front of you or you see someone smoking?
① I find smokers attractive ③ Indifferent ② Dislike it

3. ( ) In the past 7 days, on how many days did someone smoke inside your home while you were at home? (Including guests, etc.)
① 0 days ② 1‑2 days ③ 3‑4 days ④ 5‑6 days ⑤ 7 days

4. In the past 30 days, has any family member who lives with you smoked? (Select all that apply)

| Father 4_1 | Mother 4_2 | Grandfather (paternal) 4_3 | Grandmother (paternal) 4_4 | Grandmother (maternal) 4_5 | Grandfather (maternal) 4_6 | Other (please specify) 4_7 |
| --- | --- | --- | --- | --- | --- | --- |
| Yes | □ | □ | □ | □ | □ | ________ |
| No | □ | □ | □ | □ | □ |  |

5.( ) When your family members smoke, do you advise them to quit?
① My family members do not smoke ② Yes ③ No

6. ( ) When your family members smoke at home, do you discourage them?
① My family members do not smoke ② Yes ③ No

7. ( ) Do you ask your family members to avoid you when they smoke?
① My family members do not smoke ② My family members avoid me when they smoke ③ Yes ④ No

8. In the past 30 days, have you seen anyone smoking indoors or outdoors at school? (Select all that apply)

|  | Students 8_1 8_2 | Teachers 8_3 8_4 | Other personnel 8_5 8_6 |
| --- | --- | --- | --- |
| **Indoors** | □ Yes □ No | □ Yes □ No | □ Yes □ No |
| **Outdoors** | □ Yes □ No | □ Yes □ No | □ Yes □ No |

9. ( ) Do any of your friends (classmates) smoke cigarettes?
   ① Yes ② No
10. ( ) Do your friends (classmates) smoke in front of you?
    ① My friends (classmates) do not smoke ② Yes ③ No
11. ( ) When your friends (classmates) smoke cigarettes, do you advise them to quit?
    ① My friends (classmates) do not smoke ② Yes ③ No

V. Third-hand Smoke Exposure
(Third-hand smoke refers to the tobacco residue left behind after a smoker exhales, which clings to clothes, walls, carpets, furniture, and even hair and skin.)

1. ( ) Before completing this questionnaire, did you know what thirdhand smoke is?
① Yes ② No

2. ( ) In the past week, have you smelled smoke odor on furniture, clothing, carpets, or other items in your home?
① Yes ② No

VI. E‑cigarette Use

1. ( ) Have you ever used e‑cigarettes?
① Yes ② No

2. ( ) What is the main reason for your e‑cigarette use?
① I have never used e‑cigarettes ② Curiosity ③ To help quit smoking ④ Believe e‑cigarettes are less harmful ⑤ Restricted by tobacco control policies ⑥ Others around me use them ⑦ Think it is fashionable ⑧ Other, please specify ______

3. ( ) Do you think you will use e‑cigarettes in the next 12 months?
① Yes ② No

4. ( ) Do any of your friends (classmates) use e‑cigarettes?
① Yes ② No

5. How much money do you spend on e‑cigarettes per month on average?
① I have never used e‑cigarettes ② I spend ______ yuan

VII. Knowledge and Perceptions

1. ( ) Do you think smoking cigarettes is fashionable?
① Yes ② No

2. ( ) Do you think it is easy to quit once you start smoking cigarettes?
① Yes ② No

3. ( ) Do you believe that secondhand smoke from cigarettes can cause the following health harms?

| Health condition | Yes | No |
| --- | --- | --- |
| Cardiovascular diseases (coronary heart disease, hypertension, stroke, etc.) | □ | □ |
| Respiratory diseases (chronic bronchitis, emphysema, etc.) | □ | □ |
| Cancers (lung cancer, oral cancer, laryngeal cancer, etc.) | □ | □ |
| Reduced immunity | □ | □ |
| Digestive problems | □ | □ |
| Reproductive system problems | □ | □ |
| Skin aging | □ | □ |
| Dental problems | □ | □ |
| Osteoporosis | □ | □ |
| Psychological problems (anxiety, depression, etc.) | □ | □ |

4. ( ) Do you think thirdhand smoke is harmful to health?
   ① Yes ② No ③ Do not know
5. ( ) Do you think adolescents can become addicted to e‑cigarettes?
   ① Yes ② No ③ Do not know
6. ( ) Do you think using e‑cigarettes is attractive?
   ① Yes ② No
7. ( ) Do you think it is easy to quit e‑cigarettes?
   ① Yes ② No
8. Do you think e‑cigarettes contain nicotine?
   ① Some contain, some do not ② Do not contain nicotine ③ Contain nicotine ④ Do not know
9. ( ) Do you think the aerosol from e‑cigarettes contains harmful substances?
   ① Yes ② No ③ Do not know
10. In the past 30 days, how many times have you talked with friends about smoking‑related topics? (including conversations about encouraging quitting, etc.)
    Total ______ times.
11. In the past 30 days, how many times have you talked with family members about smoking‑related topics? (including conversations about encouraging quitting, etc.)
    Total ______ times.

VIII. Attitudes toward Activities

1. How much do you support the following tobacco control activity formats? (Select all that apply)

| Activity format | Strongly support | Support | Do not support |
| --- | --- | --- | --- |
| Brochures | □ | □ | □ |
| Lectures | □ | □ | □ |
| Knowledge competitions | □ | □ | □ |
| Peer games | □ | □ | □ |
| WeChat posts | □ | □ | □ |
| Short videos | □ | □ | □ |
| Online quizzes/mini‑games | □ | □ | □ |
| Animated mini‑game showing facial effects of smoking | □ | □ | □ |

2. ( ) Do you have any other comments or suggestions?
   ① No ② Yes (please specify) __________________________________________

Thank you for participating in this survey!

Interviewer's signature: ________ Quality control officer's signature: _______

Date of interview: __________ Date of quality control: __________
