# Supplementary material for: School-Family Linked and Online-Offline Combined Tobacco Control Intervention for Adolescents: Protocol for a Cluster Randomized Pilot Trial
Source: JMIR Res Protoc. 2026 Oct 21;15:e86503. doi: 10.2196/86503 (PMC13686337; doi:10.2196/86503)
Supplement: Supplementary file 3 [file resprot-v15-e86503-s002.docx]

**Student Qualitative Questionnaire**

**Intervention Measures**

1. (Look at the activity list) Have all these activities been carried out? (Were they implemented as planned?)

Probe: If not all were completed, which activities were not carried out? Why were they not carried out?

2. Please briefly recall what tobacco‑control‑related knowledge you have learned through the activities so far. (What are some things you did not know before but now know?)
3. When were the activities usually held? How many times were they held during breaks? How many times during class? Did they make you feel tired?
4. Do you think the frequency of distributing brochures, sending short videos, giving lectures, conducting peer games, holding knowledge contests, and pushing online quiz games was appropriate? Is there anything that could be improved?
5. What improvements do you think could be made to the content of the lectures, peer games, and knowledge contests?
6. The "Passport" (activity booklet)
   (1) Was such a booklet ever distributed?
   Probe: If yes, when was it distributed? If no, did the teacher explain why it was not distributed?
   (2) Did you complete all the questions in it? Were you willing to do these questions? Did your parents grade them?
    Probe: If not completed, was it because there were too many questions, they were too difficult, or there was not enough time?
    Probe: Why were you willing / unwilling? Which questions did you like, and which did you dislike?
    Probe: If they were graded, how did your parents grade them? What did they say? If not graded, was it because your parents were too busy or they thought it was unnecessary?
   (3) What additional content do you think could be added to this booklet?
   (4) Did the teacher tell you how to use this booklet? (For stamp exchanges)
7. How many brochures/flyers did you receive in total? Did you read them? Did your parents read them together with you? Did your parents think it was necessary to read them?
    Probe: If you read them, what content do you remember? If you did not read them, why not?
    Probe: If you read them together, how did your parents engage with you? What did they say? If not together, was it because your parents had no time or they did not want to read them?
    Probe: What reasons did your parents give for thinking it was necessary or unnecessary? What did they say?
8. How many tobacco‑control short videos were sent to you? Did you watch them? Did your parents watch them with you? Did your parents think it was necessary to watch them?
    Probe: If you watched them, which one did you like most? What did you remember? If you did not watch them, why not?
    Probe: If you watched together, how did your parents comment? If not together, was it because your parents did not receive them or they did not want to watch?
    Probe: What reasons did your parents give for thinking it was necessary or unnecessary?
9. Mini‑games
   (1) Did your parents receive such mini‑games on their phones?
   Probe: If yes, did your parents let you play? What did they say? If not, what do you think might be the reason?
   (2) How many times did you play? Did your parents play with you?
   Probe: If you played together, how did your parents participate? How did you play? If not together, was it because your parents did not allow it, or you did not want to play with them?
   (3) What improvements do you think could be made to the mini‑games?
10. Are you willing to cooperate with the teacher to carry out the activities as required? (Compliance)
11. Please rank the activities you have participated in according to how much you liked them, and explain the reasons. (Satisfaction)
12. What comments have your parents made about the activities? (Did they complain? Did they praise?)

**External Environment**

1. Smoke‑free campus: As far as you know, has the school previously organised similar tobacco‑control activities? Do you think teachers and parents are highly supportive of the activities? Do you know that smoking is not allowed on campus?
    Probe: If yes, what activities were they? In which grade were they held? Which did you think was better, this one or the previous ones?
    Probe: How supportive do you think they are? What specific things show that?
    Probe: If you knew, where did you learn it? If you did not know, now that we have told you, would you remind others?
2. Smoke‑free family: Have you ever heard of a smoke‑free family before? (If not, briefly explain.) Do you support the creation of smoke‑free families? Does your community offer any rewards or incentives for families that participate in establishing a smoke‑free family?
    Probe: If you have heard of it, what did you understand a smoke‑free family to be? Was it the same as described in this activity?
    Probe: Why do you support / not support it? Do you think it is difficult to achieve?
    Probe: If there are rewards, what have you heard about them? If not, what kind of rewards would you hope for?

**Implementation and Sustainability Infrastructure**

1. What kind of rewards did you receive in activities such as the knowledge contest? (Financial/material support)
2. During the distribution of brochures, lectures, peer games, and knowledge contests, how were the venues? How was the order/discipline? (Human resource support)
3. Were the lecturers who gave the talks able to deliver them smoothly? Were they able to answer everyone's questions well? (Training situation)
4. Did the teachers and staff encourage you to complete the activity content? Did they remind you frequently, sometimes, or only once when the materials were distributed?

**Homeroom Teacher Interview Guide**

1. What was your main role in the tobacco‑control intervention carried out by the school last semester?

2. (Show the activity list to the teacher) Which of these activities were not carried out? Why? (Reasons for not following the plan)

3. Approximately how many students or parents signed the consent form with a "no"? Do you know why they chose not to participate?

4. Were there any students who did not follow your or the staff's instructions? Why? How was it handled afterwards?

5. Do you think the overall frequency of the activities was appropriate? What could be improved?

6. Did the staff from the District CDC provide any guidance? Were all the lectures delivered by the District CDC personnel? (Compliance)

7. Do you think this guidance was necessary? Are there any other areas where guidance is needed?

8. Did you maintain order during the activities? If so, was it challenging? (Human resource support)

9. Did you receive any training before participating in the activities? When and where was the training held? What was the content? Who provided the training? Were the trainers able to answer all the questions thoroughly? (Training situation)

10. Did you see any leaders or staff conducting supervision visits while you were participating in or organising the activities? In what form? (Supervision situation)

11. Did you encourage or remind parents and children to read the brochures together, watch the short videos, and complete the online games and quiz mini‑games? Did you remind them frequently, occasionally, or only once when the materials were pushed?

12. Were there any complaints from parents about the activities that led to complaints to the school or teachers? What were the complaints about? How were they handled afterwards?

13. Please rank the activities you have participated in according to how much you liked them, and explain the reasons. (Satisfaction)

14. Has the school previously organised similar tobacco‑control activities? ① Are you supportive of this activity? ② Are other teachers supportive of this activity?

15. In the past three months, did any of your colleagues smoke around you? If so, did you advise them to quit? How did you do that?

16. In the past three months, did any of your family members smoke around you? If so, did you advise them to quit? How did you do that?

17. In the past three months, did any of your friends smoke around you? If so, did you advise them to quit? How did you do that?

18. Is there a smell of smoke in any hidden corners of the school?

**School Nurse Interview Guide**

1. What role did you play in the tobacco‑control intervention carried out during the last semester?

2. Did the students and parents participate as planned? Approximately how many people did not participate as planned? Do you know the reasons why they did not participate?

3. Were you aware that some procedures or steps were modified to make the activity process and procedures easier to implement? Please give examples. (Compliance)

4. Were there any students who did not follow your or the staff's instructions? Why? How was it handled afterwards? (Compliance)

5. Were there any complaints from parents about the activities that led to complaints to the school or teachers? What were the complaints about? How were they handled afterwards?

6. Please take a look: have all these activities been carried out?

Probe: Do you know why the XX activity was cancelled? (Reasons for not following the plan)

7. Do you think the overall frequency of the activities was appropriate? What changes would make it more suitable? (Frequency)

8. What improvements do you think could be made to the overall content of the activities? (Content)

9. Did you carry out these activities on your own, or did you have a team to assist you? Do you think these intervention activities took away time that you would otherwise have spent on handling daily health issues for the students?

10. Did the school provide any additional financial support for this activity? If so, what aspects were funded? (Teacher compensation? Purchase of small gifts? …)

11. Were the activities held in the classrooms or in the auditorium? Who was responsible for coordinating the venue? Did you provide any assistance in the venue coordination?

Probe: If yes, what specific help did you provide? If not, who was the main coordinator? Were there any difficulties in the coordination process?

12. Were the activities usually held during break time or during class time? Who was responsible for coordinating the schedule? How was it coordinated?

13. Were you responsible for the staffing arrangements for organising and implementing the activities? If so, were everyone satisfied with your arrangements? Did anyone express dissatisfaction?

14. Did you provide any training to the teachers?

Probe: If yes, how many training sessions were held? What was the main content? How did the teachers respond? If not, why was there no training? Did the teachers have any difficulties starting directly?

15. Please rank the activities you have participated in according to how much you liked them, and explain the reasons. (Satisfaction)

16. In the past three months, did any of your colleagues smoke around you? If so, did you advise them to quit? How did you do that?

17. In the past three months, did any of your family members smoke around you? If so, did you advise them to quit? How did you do that?

18. In the past three months, did any of your friends smoke around you? If so, did you advise them to quit? How did you do that?

19. Is there a smell of smoke in any hidden corners of the school?

Probe: If yes, where roughly? How often do you smell it? Have you taken any measures? If not, how does the school manage to prevent it?

**CDC Staff Interview Guide**

1. What role did you play in the overall tobacco‑control project? (Director? Implementer?)

2. Did you carry out the activities according to the guidance provided by the Municipal CDC staff?

Probe: If not all guidance was followed, which aspects were adjusted? Why were they adjusted? Did you communicate with the Municipal CDC before making the adjustments?

3. Were any activities cancelled by individual schools? If so, do you know the reasons for the cancellation? (Reasons for not following the plan)

4. Do you think the overall frequency of the activities was appropriate?

Probe: If not, which specific activities need their frequency adjusted? Did the schools give any feedback about the frequency?

5. Did the students, school nurses, homeroom teachers, and other staff cooperate during the activities? If not, how was the communication and handling done afterwards? (Compliance)

6. Were there any complaints from parents about the activities that led to complaints to your organisation? What were the complaints about? How were they handled afterwards?

7. Please rank the activities you have participated in according to how much you liked them, and explain the reasons. (Satisfaction)

8. Do any of your colleagues smoke at work? Do they smoke indoors? Do you advise them to quit? How do you do that?

9. Does your organisation have a smoke‑free policy?

Probe: If yes, is the policy strictly enforced? Are there any rewards or penalties? If not, why hasn't such a policy been established?

10. Did your organisation provide any additional material resources for this tobacco‑control activity?

Probe: If yes, what exactly was provided? If not, was it due to budget constraints or because it was deemed unnecessary? How much did this affect the implementation of the activities?

11. How many people from your organisation participated in this activity? Approximately what proportion? Which activities did they take part in?

12. Did you provide any secondary training to the participating staff or school teachers? What content was covered in the training? (Training situation)

13. Did you conduct quality control checks on the school activities? (Supervision situation)

Probe: If yes, how was the quality control carried out? How often? What problems were identified? If not, why wasn't quality control performed? How was the quality of the activities ensured?
