# Supplementary material for: School-Family Linked and Online-Offline Combined Tobacco Control Intervention for Adolescents: Protocol for a Cluster Randomized Pilot Trial
Source: JMIR Res Protoc. 2026 Oct 21;15:e86503. doi: 10.2196/86503 (PMC13686337; doi:10.2196/86503)
Supplement: Supplementary file 4 [file resprot-v15-e86503-s003.docx]

**Sample Size**

For experiments with four equally balanced parallel groups (three intervention arms and one control), sample size estimation was performed using the formula for multiple group comparisons:。

In this formula, p_0_ denotes the expected awareness rate of smoking-related diseases in the control group; p_1_ denotes the expected rate in the intervention groups, calculated as.

OR is the odds ratio for the intervention relative to the control, for which we adopted a conservative estimate.

Based on a previous school-based tobacco control study, the awareness rate of tobacco-related diseases in the control group was 90.6%, while that in the intervention group was 99.3%, yielding an OR of approximately 0.75. Assuming a cluster size of 40 students per class, a two-sided significance level of α=0.05, and statistical power of 1-β=0.90, the required number of clusters was 4 per group, corresponding to 160 students per group (640 in total). To account for the design effect due to cluster randomization, we calculated, where m=40 is the average cluster size and the intracluster correlation coefficient was conservatively estimated at 0.02. This yielded a deff of 1.78.

### Statistical Analysis

Let denote the outcome variable for the i‑th individual at the t‑th measurement ( for baseline, for endline). This study constructs the following marginal model:

where is the marginal expectation of the outcome variable; g is the link function, selected according to the type of outcome variable: a logit link is used for binary outcomes (e.g., whether thirdhand smoke is considered harmful), and an identity link is used for continuous outcomes (e.g., composite score of knowledge regarding diseases caused by secondhand smoke); is a time dummy variable (0 = baseline, 1 = endline); is a group dummy variable, with the control group as the reference, and dummy variables are set for the online intervention group, offline intervention group, and combined online‑offline intervention group; is the time‑by‑group interaction term, whose coefficient β₃ is the parameter of primary interest, representing the net effect of each intervention group relative to the control group; is the vector of covariates, including confounding factors such as gender, region of residence, family co‑habitation status, and personality traits; γ is the vector of regression coefficients for the covariates.
