# Supplementary material for: School-Family Linked and Online-Offline Combined Tobacco Control Intervention for Adolescents: Protocol for a Cluster Randomized Pilot Trial
Source: JMIR Res Protoc. 2026 Oct 21;15:e86503. doi: 10.2196/86503 (PMC13686337; doi:10.2196/86503)
Supplement: Supplementary file 5 [file resprot-v15-e86503-s004.docx]

Table. Implementation evaluation

| PRISM dimension | Recipients perspective | Organizational perspective | Implementation and Sustainability Infrastructure | External  Environment |
| --- | --- | --- | --- | --- |
| Recipients centeredness | Focus on students' interests and safety. |  |  |  |
| Provides recipients choices | Students have the right to choose to withdraw freely. |  |  |  |
| Addresses recipients barriers | Provide project explanation before the start of the project. |  |  |  |
| Service and access | Students receive intervention materials and participate in activities. |  |  |  |
| Burden (complexity and cost) | Students receive gifts. |  |  |  |
| Feedback of results | Questionnaires and interviews |  |  |  |

Table (continued).Implementation evaluation

| Readiness |  | Conduct a project kickoff meeting and prepare materials. |  |  |
| --- | --- | --- | --- | --- |
| Addresses barriers of frontline staff |  | Resolve staff issues in a timely manner through the WeChat group. |  |  |
| Coordination across departments and specialties |  | Invite experts from relevant fields to discuss the questionnaire and interview content. |  |  |
| Burden (complexity and cost) |  | Inquire about the additional expenses incurred by each department during the intervention. |  |  |
| Usability and adaptability |  | Conduct interviews with interviews project with implementation the personnel project. implementation personnel. |  |  |

Table (continued).Implementation evaluation

| Dedicated team |  |  | Composed of teachers and students from the China CDC, Beijing CDC, and Capital Medical University. |  |
| --- | --- | --- | --- | --- |
| Adopter training and support |  |  | Training was provided before the implementation of the project. |  |
| Relationship and communication with adopters (bridge researchers) |  |  | Teachers from the Beijing CDC and the CDCs of various districts. |  |
| Plan for sustainability |  |  | The materials and mini-programs from the project will be promoted after the project concludes. |  |
| Regulatory environment |  |  |  | Beijing already has smoking control regulations. |

*CDC*: Centers for Disease Control and Prevention
