# Supplementary material for: School-Family Linked and Online-Offline Combined Tobacco Control Intervention for Adolescents: Protocol for a Cluster Randomized Pilot Trial
Source: JMIR Res Protoc. 2026 Oct 21;15:e86503. doi: 10.2196/86503 (PMC13686337; doi:10.2196/86503)
Supplement: Supplementary file 6 [file resprot-v15-e86503-s005.docx]

Table 1. Sociodemographic Characteristics

| Variable | Category | Control group (%) (n=327) | Online intervention group (%) (n=319) | Offline intervention group (%) (n=324) | Combined intervention group (%) (n=331) |
| --- | --- | --- | --- | --- | --- |
| Gender | Male | 165 (50.5) | 152 (47.6) | 167 (51.5) | 160 (48.3) |
|  | Female | 162 (49.5) | 167 (52.4) | 157 (48.5) | 171 (51.7) |
| Living arrangement | Not living with parents | 2 (0.6) | 3 (0.9) | 3 (0.9) | 12 (3.6) |
|  | Living with both parents | 287 (87.8) | 283 (88.7) | 281 (86.7) | 273 (82.5) |
|  | Living with father only | 12 (3.7) | 7 (2.2) | 9 (2.8) | 15 (4.5) |
|  | Living with mother only | 26 (8.0) | 26 (8.2) | 31 (9.6) | 31 (9.4) |
| Permanent residence | Fengtai | 114 (34.9) | 111 (34.8) | 115 (35.5) | 112 (33.8) |
|  | Tongzhou | 109 (33.3) | 110 (34.5) | 118 (36.4) | 114 (34.4) |
|  | Fangshan | 104 (31.8) | 98 (30.7) | 91 (28.1) | 105 (31.7) |

 Table 2. Personality Traits

| Variable | Category | Control group (%) (n=327) | Online intervention group (%) (n=319) | Offline intervention group (%) (n=324) | Combined intervention group (%) (n=331) |
| --- | --- | --- | --- | --- | --- |
| Extraversion | High-score group | 225 (17.29) | 206 (15.83) | 233 (17.91) | 214 (16.45) |
|  | Low-score group | 102 (7.84) | 113 (8.69) | 91 (6.99) | 117 (8.99) |
| Agreeableness | High-score group | 246 (18.91) | 242 (18.60) | 268 (20.60) | 240 (18.45) |
|  | Low-score group | 81 (6.23) | 77 (5.92) | 56 (4.30) | 91 (6.99) |
| Conscientiousness | High-score group | 174 (13.37) | 186 (14.30) | 187 (14.37) | 178 (13.68) |
|  | Low-score group | 153 (11.76) | 133 (10.22) | 137 (10.53) | 153 (11.76) |

Table 2 (continue). Personality Traits

| Variable | Category | Control group (%) (n=327) | Online intervention group (%) (n=319) | Offline intervention group (%) (n=324) | Combined intervention group (%) (n=331) |
| --- | --- | --- | --- | --- | --- |
| Emotional Stability | High-score group | 90 (6.92) | 95 (7.30) | 91 (6.99) | 85 (6.53) |
|  | Low-score group | 237 (18.22) | 224 (17.22) | 233 (17.91) | 246 (18.91) |
| Openness | High-score group | 103 (7.92) | 214 (16.45) | 220 (16.91) | 193 (14.83) |
|  | Low-score group | 224 (17.22) | 105 (8.07) | 104 (7.99) | 138 (10.61) |

Table 3. Cigarette Use Characteristics

| Variable | Category | Control group (%) (n=327) | Online intervention group (%) (n=319) | Offline intervention group (%) (n=324) | Combined intervention group (%) (n=331) |
| --- | --- | --- | --- | --- | --- |
| Cigarette use in the past 30 days | 0 days | 321 (98.2) | 312 (97.8) | 320 (98.8) | 325 (98.2) |
|  | ≥1 day | 6 (1.8) | 7 (2.2) | 4 (1.2) | 6 (1.8) |
| Average daily cigarettes smoked | 0 cigarettes | 321 (98.2) | 317 (99.4) | 322 (99.4) | 327 (98.8) |
|  | >0 cigarettes | 6 (1.8) | 2 (0.6) | 2 (0.6) | 4 (1.2) |
| Intention to smoke in the next 12 months | Yes | 5 (1.5) | 7 (2.2) | 8 (2.5) | 6 (1.8) |
|  | No | 322 (98.5) | 312 (97.8) | 316 (97.5) | 325 (98.2) |

Table 4. Secondhand Smoke Exposure

| Variable | Category | Control group (%) (n=327) | Online intervention group (%) (n=319) | Offline intervention group (%) (n=324) | Combined intervention group (%) (n=331) |
| --- | --- | --- | --- | --- | --- |
| Reaction to cigarette smoke odor | Like | 4 (1.2) | 5 (1.6) | 8 (2.5) | 7 (2.1) |
|  | Dislike | 267 (81.7) | 270 (84.6) | 250 (77.2) | 267 (80.7) |
|  | Indifferent | 56 (17.1) | 44 (13.8) | 66 (20.4) | 57 (17.2) |
| Reaction when someone smokes in front of you | Find smokers attractive | 1 (0.3) | 4 (1.3) | 3 (0.9) | 2 (0.6) |
|  | Dislike | 238 (72.8) | 246 (77.1) | 227 (70.1) | 241 (72.8) |
|  | Indifferent | 88 (26.9) | 69 (21.6) | 94 (29.0) | 88 (26.6) |
| Number of days someone smoked at home in the past 7 days | 0 days | 191 (58.4) | 180 (56.4) | 177 (54.6) | 207 (62.5) |
|  | 1–2 days | 45 (13.8) | 49 (15.4) | 47 (14.5) | 50 (15.1) |
|  | 3–4 days | 24 (7.3) | 28 (8.8) | 27 (8.3) | 10 (3.0) |
|  | 5–6 days | 7 (2.1) | 13 (4.1) | 16 (4.9) | 10 (3.0) |
|  | 7 days | 60 (18.3) | 49 (15.4) | 57 (17.6) | 54 (16.3) |

Table 4 (continue). Secondhand Smoke Exposure

| Variable | Category | Control group (%) (n=327) | Online intervention group (%) (n=319) | Offline intervention group (%) (n=324) | Combined intervention group (%) (n=331) |
| --- | --- | --- | --- | --- | --- |
| Whether you discourage family members from smoking | My family members do not smoke | 111 (33.9) | 113 (35.4) | 103 (31.8) | 105 (31.7) |
|  | Yes | 169 (51.7) | 173 (54.2) | 177 (54.6) | 191 (57.7) |
|  | No | 47 (14.4) | 33 (10.3) | 44 (13.6) | 35 (10.6) |
| Whether you discourage family members from smoking at home | My family members do not smoke | 118 (36.1) | 117 (36.7) | 110 (34.0) | 113 (34.1) |
|  | Yes | 166 (50.8) | 175 (54.9) | 166 (51.2) | 180 (54.4) |
|  | No | 43 (13.1) | 27 (8.5) | 48 (14.8) | 38 (11.5) |

Table 4 (continue). Secondhand Smoke Exposure

| Variable | Category | Control group (%) (n=327) | Online intervention group (%) (n=319) | Offline intervention group (%) (n=324) | Combined intervention group (%) (n=331) |
| --- | --- | --- | --- | --- | --- |
| Whether you ask family members to avoid you when they smoke | My family members do not smoke | 115 (35.2) | 116 (36.4) | 108 (33.3) | 115 (34.7) |
|  | My family members avoid me when they smoke | 130 (39.8) | 128 (40.1) | 126 (38.9) | 145 (43.8) |
|  | Yes (I ask them to) | 40 (12.2) | 46 (14.4) | 53 (16.4) | 36 (10.9) |
|  | No | 42 (12.8) | 29 (9.1) | 37 (11.4) | 35 (10.6) |
| When your friends (classmates) smoke cigarettes, do you advise them to quit? | My friends (classmates) do not smoke | 252 (77.1) | 271 (85.0) | 283 (87.3) | 268 (81.0) |
|  | Yes | 46 (14.1) | 29 (9.1) | 29 (9.0) | 38 (11.5) |
|  | No | 29 (8.9) | 19 (6.0) | 12 (3.7) | 25 (7.6) |

Table 5. Third-hand Smoke Exposure

| Variable | Category | Control group (%) (n=327) | Online intervention group (%) (n=319) | Offline intervention group (%) (n=324) | Combined intervention group (%) (n=331) |
| --- | --- | --- | --- | --- | --- |
| Whether there was a smoke odor on furniture, clothing, carpets, etc. at home in the past week | Yes | 86 (26.3) | 56 (17.6) | 63 (19.4) | 71 (21.5) |
|  | No | 241 (73.7) | 263 (82.4) | 261 (80.6) | 260 (78.5) |

Table 6. E-cigarette Use

| Variable | Category | Control group (%) (n=327) | Online intervention group (%) (n=319) | Offline intervention group (%) (n=324) | Combined intervention group (%) (n=331) |
| --- | --- | --- | --- | --- | --- |
| Intention to use e-cigarettes in the next 12 months | Yes | 3 (0.9) | 8 (2.5) | 3 (0.9) | 4 (1.2) |
|  | No | 324 (99.1) | 316 (97.5) | 324 (99.1) | 327 (98.8) |

Table 7. Tobacco-Related Knowledge and Perceptions

| Variable | Category | Control group (%) (n=327) | Online intervention group (%) (n=319) | Offline intervention group (%) (n=324) | Combined intervention group (%) (n=331) |
| --- | --- | --- | --- | --- | --- |
| Do you think smoking cigarettes is fashionable? | Yes | 8 (2.4) | 5 (1.6) | 4 (1.2) | 11 (3.3) |
|  | No | 319 (97.6) | 314 (98.4) | 320 (98.8) | 320 (96.7) |
| Is it easy to quit once you start smoking cigarettes? | Yes | 35 (10.7) | 34 (10.7) | 37 (11.4) | 42 (12.7) |
|  | No | 292 (89.3) | 285 (89.3) | 287 (88.6) | 289 (87.3) |
| Comprehensive score of knowledge about secondhand smoke causing diseases | Mean ± SD | 1.79±0.27 | 1.78±0.26 | 1.76±0.28 | 1.83±0.25 |
| Do you think thirdhand smoke is harmful to health? | Yes | 274 (83.8) | 268 (84.0) | 266 (82.1) | 265 (80.1) |
|  | No | 11 (3.4) | 24 (7.5) | 14 (4.3) | 20 (6.0) |
|  | Do not know | 42 (12.8) | 27 (8.5) | 44 (13.6) | 46 (13.9) |

Table 7 (continue). Tobacco-Related Knowledge and Perceptions

| Variable | Category | Control group (%) (n=327) | Online intervention group (%) (n=319) | Offline intervention group (%) (n=324) | Combined intervention group (%) (n=331) |
| --- | --- | --- | --- | --- | --- |
| Do you think adolescents can become addicted to e‑cigarettes? | Yes | 256 (78.3) | 242 (75.9) | 258 (79.6) | 243 (73.4) |
|  | No | 8 (2.4) | 7 (2.2) | 6 (1.9) | 15 (4.5) |
|  | Do not know | 63 (19.3) | 70 (21.9) | 60 (18.5) | 73 (22.1) |
| Do you think e‑cigarettes are attractive? | Yes | 14 (4.3) | 9 (2.8) | 11 (3.4) | 10 (3.0) |
|  | No | 313 (95.7) | 310 (97.2) | 313 (96.6) | 321 (97.0) |
| Do you think it is easy to quit e‑cigarettes? | Yes | 31 (9.5) | 28 (8.8) | 44 (13.6) | 48 (14.5) |
|  | No | 296 (90.5) | 291 (91.2) | 280 (86.4) | 283 (85.5) |

Table 7 (continue). Tobacco-Related Knowledge and Perceptions

| Variable | Category | Control group (%) (n=327) | Online intervention group (%) (n=319) | Offline intervention group (%) (n=324) | Combined intervention group (%) (n=331) |
| --- | --- | --- | --- | --- | --- |
| Do you think e‑cigarettes contain nicotine? | Some contain, some do not | 103 (31.5) | 72 (22.6) | 79 (24.4) | 110 (33.2) |
|  | Do not contain nicotine | 9 (2.8) | 6 (1.9) | 11 (3.4) | 6 (1.8) |
|  | Contain nicotine | 88 (26.9) | 96 (30.1) | 91 (28.1) | 72 (21.8) |
|  | Do not know | 127 (38.8) | 145 (45.5) | 143 (44.1) | 143 (43.2) |
| Do you think the aerosol from e‑cigarettes contains harmful substances? | Yes | 255 (78.0) | 248 (77.7) | 240 (74.1) | 249 (75.2) |
|  | No | 6 (1.8) | 7 (2.2) | 11 (3.4) | 9 (2.7) |
|  | Do not know | 66 (20.2) | 64 (20.1) | 73 (22.5) | 73 (22.1) |
